# Idiopathic Prolonged Long QT Interval Leading to Sudden Cardiac Arrest in an Adolescent

**DOI:** 10.7759/cureus.71321

**Published:** 2024-10-12

**Authors:** Bianca Elias, Mazen Zamzam, Hashem Mohilldean, Kenan Sinan, Ibrahim Kassas

**Affiliations:** 1 Internal Medicine, Oakland University William Beaumont School of Medicine, Rochester, USA; 2 Internal Medicine, Wayne State University School of Medicine, Detroit, USA; 3 Interventional Cardiology, Advocate Christ Medical Center, Oak Lawn, USA

**Keywords:** arrhythmia, cardiac arrest adolescent, long qt, sudden cardiac arrest, syncope

## Abstract

Sudden cardiac arrest (SCA) can be caused by inherited and acquired conditions and can lead to sudden cardiac death. There are many causes and risk factors for SCA such as QTc prolongation which can be inherited or acquired. Potential causes of acquired QTc prolongation include medications, electrolyte abnormalities, etc. Genetic causes are less common and look at channelopathies involved in the cardiac action potential that interferes with the repolarization, causing a prolonged ventricular contraction reflected in the QTc interval. Before determining that the cause of an SCA is due to a QTc prolonged interval, a full cardiac workup needs to be done to eliminate other anatomical causes of SCA. This case report elicits an interesting clinical scenario in which a previously healthy 20-year-old male experiences SCA while playing basketball. The patient was resuscitated at the scene. After defibrillation, intubation, various imaging studies, and a subcutaneous implantable cardioverter-defibrillator (ICD) placement, the patient was diagnosed with long QTc syndrome and underwent genetic testing which was later found to be negative. Hence, understanding the causes of SCA in young patients can be important in avoiding dreadful and life-threatening situations and providing information to the patient and family members.

## Introduction

Sudden cardiac arrest (SCA) is a sudden cessation of circulation that is mostly caused by a cardiac arrhythmia that results from a cardiovascular cause. SCA accounts for 15%-20% of all natural deaths in adults in the USA [1]. SCA results from a complex interaction of inherited and acquired causes [2]. In the majority of cases, an SCA episode occurs in the community, known as an out-of-hospital cardiac arrest (OHCA) [3]. Risk factors include male gender, increased age, and African American ethnicity. The most common underlying cause of SCA is coronary artery disease (CAD), and the risk factors for CAD include diabetes, dyslipidemia, cigarette smoking, obesity, and hypertension. This also applies to younger individuals. According to a report from the Oregon Sudden Cardiac Death (SCD) Study, 58% of patients aged 5-34 had at least one of the abovementioned congestive heart failure (CHF) risk factors and 39% were obese [4]. Genetic risk factors contribute mostly to cardiomyopathies and primary electrical disorders [2].

Specifically, in younger people, there are several causes of SCA and SCD, some of which include sudden arrhythmic death syndrome/primary electrical disorders, cardiomyopathies, coronary artery abnormalities, myocarditis-related pathology, and aortic dissection [2,3]. Diagnoses that are represented in the primary electrical disorder category include channelopathy including long QT syndrome (LQTS) and Brugada syndrome, catecholaminergic polymorphic ventricular tachycardia, and conduction abnormalities like Wolff Parkinson-White syndrome. Cardiomyopathy includes diagnoses like hypertrophic cardiomyopathy, arrhythmogenic cardiomyopathy, and dilated cardiomyopathy [3]. The key objective of this case report is to look at how a prolonged QTc interval can cause SCA and the causes of it.

## Case presentation

This is a 20-year-old male with no significant past medical history presented to the emergency department directly after he had a cardiac arrest while playing basketball. Of note, the patient reports a history of daily tobacco vaping that had increased in frequency for a short time period before his cardiac arrest. He also reports smoking marijuana daily. He reports no alcohol consumption and reports a fairly regular exercise program. This patient’s family history includes no known cardiac concerns. Shortly after arrest, a bystander started cardiopulmonary resuscitation (CPR), and on arrival by EMS, the patient was noted to be in ventricular fibrillation, and 200 joules shock was administered, return of spontaneous circulation (ROSC) was achieved, no medications were given. The suspected downtime was 8-10 minutes. The patient was intubated due to his Glasgow coma scale score being less than 8. After intubation, the patient started experiencing seizure-like activity, suspected to be in status epilepticus, and started on levetiracetam. The patient was also started on piperacillin-tazobactam for concern of potential infection of unknown origin. Vitals upon presentation demonstrated readings of blood pressure at 108/46 mmHg, pulse of 77 beats per minute, temperature of 37.3°C, respiratory rate of 19 breaths per minute, SpO_2 _of 96%, and BMI of 28.00 kg/m² 

Initial labs (Table 1) on arrival were drawn and revealed bicarbonate of 15, anion gap of 22, glucose of 145, creatinine of 1.48, alanine aminotransferase (AST) of 78, and aspartate aminotransferase (ALT) of 118. Lactic acid was also drawn and was 10. Blood gas was notable for pH of 7.27, PO_2_ of 369, and base excess of negative 7. The urine drug screen was positive for cannabinoids. Troponin levels, alcohol levels, salicylate levels, and brain natriuretic peptide levels were all found to be within normal limits. Blood and urine cultures were also drawn and later found to be negative for any growth. CT scan of the head, chest, abdomen, and pelvis did not reveal any acute findings. A 12-lead echocardiogram (ECG) showed normal sinus rhythm with normal rate, intervals, and axis. No ST segment depression or elevation was observed. 

**Table 1:** Lab values and reference ranges immediately after arrival

Test	Result	Reference range
Bicarbonate (HCO_3_)	15 mEq/L	22-29 mEq/L
Anion gap	22 mEq/L	3-11 mEq/L
Glucose	145 mg/dL	70-99 mg/dL (fasting)
Creatinine	1.48 mg/dL	0.6-1.2 mg/dL
Aspartate aminotransferase (AST)	78 U/L	10-40 U/L
Alanine aminotransferase (ALT)	118 U/L	7-56 U/L
Lactic acid	10 mmol/L	0.5-2.2 mmol/L
Blood gas-pH	7.27	7.35-7.45
Blood gas-PO_2_	369 mmHg	75-100 mmHg
Blood gas-base excess	-7 mmol/L	-2 to +2 mmol/L
Troponin levels	Within normal limits	<0.04 ng/mL
Alcohol levels	Within normal limits	0 mg/dL
Brain natriuretic peptide (BNP)	Within normal limits	<100 pg/mL

**Figure 1:**
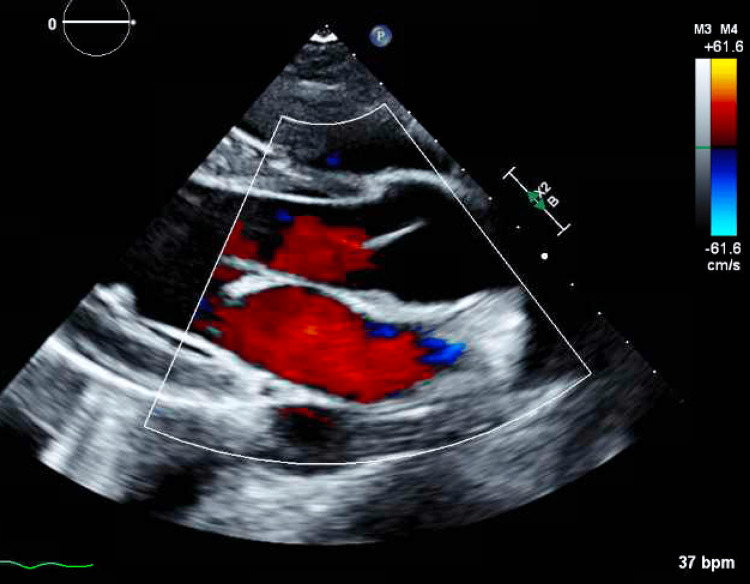
Echocardiogram (ECG) showing normal left ventricular (LV) wall thickness with an LV ejection fraction (EF) of 60%, indicating preserved systolic function

**Figure 2:**
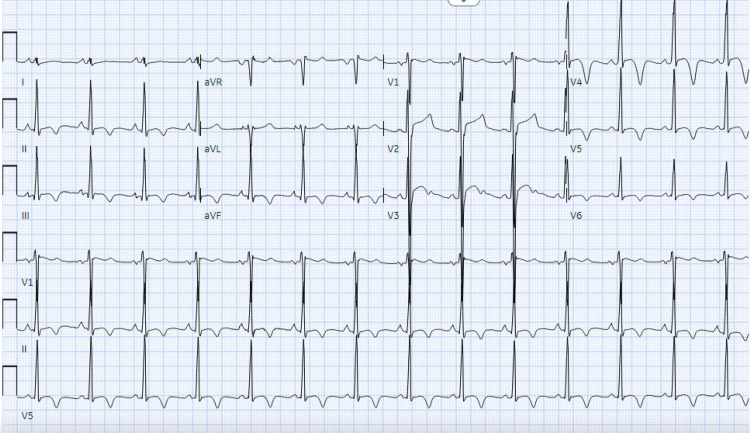
Electrocardiograms (ECGs) showing anterolateral and inferior ST-T abnormalities along with prolonged QT/QTc intervals

**Figure 3:**
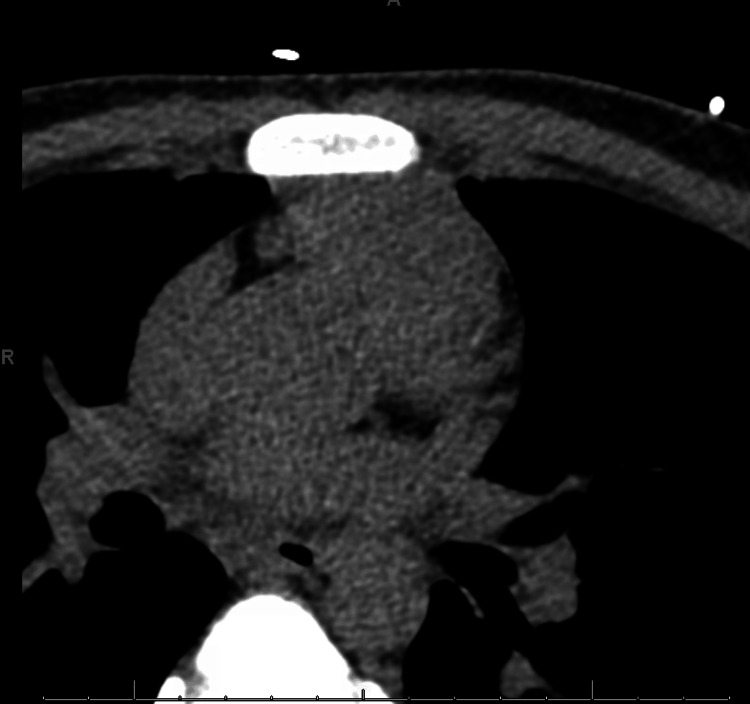
Computed tomography angiography (CTA) illustrating normal coronary artery placement, with significant left ventricular hypertrophy as well

**Figure 4:**
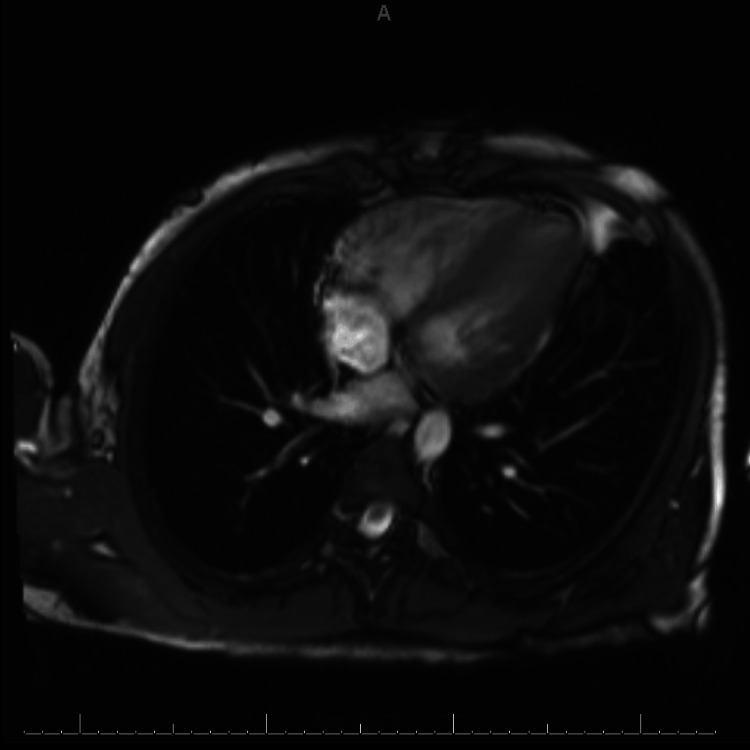
Cardiac magnetic resonance imaging (MRI) demonstrating normal left and right ventricular size and function with an ejection fraction (EF) of 61% and 65%, respectively. A small area of scar is visible in the apical anterior wall, which may be the nidus for the patient's dysrhythmia and arrest

The patient was admitted to the coronary care unit. The ECG (Figure 1) showed normal left ventricle (LV) wall thickness and an LV ejection fraction (EF) of 60%. The patient had several ECGs, with anterolateral ST-T abnormalities, inferior ST-T abnormalities, and prolonged QT/QTc found in Figure 2. CTA and cardiac MRI were obtained due to concerns of anomalous coronary artery, myocardial bridging, and hypertrophic obstructive cardiomyopathy (HCOM). CTA revealed normal coronary placement but does suggest left ventricular hypertrophy found in Figure 3. A cardiac MRI (Figure 4) was done that revealed normal left and right ventricular size and function with an EF of 61% and 65%, respectively. The cardiac MRI did reveal that a small area of scar in the apical anterior wall may be a nidus for his dysrhythmia and arrest. The patient was started on a low-dose metoprolol 12.5 mg twice daily. MRI of the brain with and without contrast was obtained to rule out cerebral ischemia and revealed no acute stroke or other acute intracranial abnormalities; therefore, levetiracetam 500 mg twice daily was discontinued. Subcutaneous implantable cardioverter-defibrillator (ICD) was placed with no complications. The patient was discharged to inpatient rehabilitation. The patient continued taking metoprolol tartrate 12.5 mg twice daily for LQTS. He was also referred for a genetic workup, which was found to be negative.

## Discussion

A QT interval represents the duration of the ventricular action potential on an ECG. This physiological correlation with the duration of the ventricular depolarization and repolarization [4]. Because the QT interval varies with heart rate, it needs to be corrected for the heart rate, and this is known as the QTc. The most common equation used to correct for heart rate is known as the Bazett formula (QTc = QT/√ RR). Although it is relatively accurate for heart rates between 60 and 100 beats/min, it tends to overcorrect with higher rates and undercorrect with lower heart rates. A QTc is prolonged if it is greater than 440 ms in men or greater than 460 ms in women. A QTc greater than 500 is associated with an increased risk of torsade de pointes, a type of polymorphic ventricular tachycardia. Torsade de pointes may degenerate into ventricular fibrillation and cause SCD [5]. This patient during the hospital stay was noted to have several ECGs with a prolonged QT and QTc interval, with the highest QTc being 477 ms. 

Congenital and acquired factors can lead to QTc prolongation. The acquired form is mostly associated with drug prolonging the interval or due to electrolyte disturbances [4]. Common medication drug classes which can prolong QTc interval include antineoplastics, antihistamines, antipsychotics, antiemetics, antimicrobials, and antiarrhythmics. Electrolyte disturbances such as hypokalemia, hypomagnesemia, and hypocalcemia can also contribute to QTc prolongation [2]. Our patient experienced a prolonged QT interval after the administration of haloperidol. It is important to note the temporal relationship between haldol administration, an antipsychotic, and the observed QT interval prolongation. 

The congenital LQTS is composed of a group of heritable conditions that are associated with cardiac repolarization dysfunction [4]. The voltage-gated potassium channel is mainly responsible for the repolarization of the cardiac myocytes and there are two subtypes Kv7.1 and Kv11.1. The genes that are involved in LQTS are caused by loss-of-function in voltage-gated potassium channels in Kv7.1 and Kv11.1 account for about 80% of all genotypes positive LQTS cases [4,5]. These involved genes known as KCNQ1, KCNH2, KCNE1, and KCNE2. There can also be defects in the sodium ion channel in genes SCN5A that can cause a delay in the plateau phase of the action potential, also causing delayed repolarization. Our patient underwent DNA genetic testing to look for all those genes and many more and was later found to have a negative result.

For long-term management of these patients with LQTS, beta-blockers are the first-line treatment. Beta-blockers help prevent ventricular arrhythmias by stabilizing ventricular action potential and help block sympathetic surges associated with arrhythmias. Patients should also undergo subcutaneous ICD placement, especially those patients who were resuscitated from a cardiac arrest, such as this patient.

## Conclusions

There are multiple causes of SCA; therefore, careful workup can help to determine the leading etiology of this disease. In our case, involving an electrophysiologist, obtaining cardiac MRI, cardiac angiography to rule out anomalous coronary artery, and HOCM as well as genetic testing were important steps to take. The workup was negative except for an ECG that showed a slightly atypical Brugada pattern and prolonged QTc interval. Educating the patient on risk factors and the potential for SCA or SCD is also important. Overall, this is a cumulative approach that requires both the physician team and the patient and their family to work together.
